# Introducing *PLoS Genetics*

**DOI:** 10.1371/journal.pgen.0010021

**Published:** 2005-07-25

**Authors:** Wayne N Frankel

On behalf of our editorial team, it is my pleasure to welcome you to *PLoS Genetics,* a new open-access journal from the Public Library of Science (PLoS). Led by an internationally recognized editorial board with broad knowledge and expertise, *PLoS Genetics* is a journal that celebrates the research of the greater genetics and genomics community. As you see in this first issue, *PLoS Genetics* is unique—publishing outstanding articles that reflect the full breadth and interdisciplinary nature of this research, all free to read and to use in your own research and teaching.

**How did *PLoS Genetics* come about?** In 2004, when PLoS asked several of us in the genetics community about a need and desire for an open-access genetics/genomics journal, I replied with a resounding “yes!” And I was not alone—others had the same reaction that the time was right for a new genetics journal of high quality. Certainly the open-access element was key—following in the public-domain spirit of genetics and genomics data release, for example, by the Human Genome Project. And creating such a journal—building on the strong experience and reputation of *PLoS Biology—*seemed an opportunity not to miss.

**What is the focus of *PLoS Genetics?*** Our mainstay is primary research articles, of which there are eleven in our inaugural issue. We strive for high-quality, original contributions from a broad sweep of the research community, the common theme being novel or incisive applications of genetics and genomics tools to address important research problems in any area of biology. The format itself is flexible; we encourage authors to be creative about the most appropriate and efficient way to present their research. Because we make this high-level research fully and immediately available on our Web site and through PubMed Central, scientists can keep current, build on these findings, and respond by submitting work of their own.

**Will *PLoS Genetics* have special features?** Yes—the prospect of which was another key to my own interest in being involved! In addition to research articles, *PLoS Genetics* offers a venue to consider important issues of the day. Our Reviews editors—Elizabeth Fisher, Nicholas Katsanis, Marcy MacDonald, and Susan Rosenberg—both invite reviews and consider submitted ones that summarize a particularly interesting, hot, or forward-looking aspect of genetics and genomics research, and include the authors' unique perspective on it. Beginning in August, we will aim to publish at least one review each month. Every few months, we will also offer a special feature article. For example, in this issue, Interviews Editor Jane Gitschier presents her one-on-one interview with statistical geneticist Neil Risch (DOI: 10.1371/journal.pgen.0010014).

*PLoS Genetics* celebrates the research of the greater genetics and genomics community.

In future issues, our front section will offer a correspondence forum—intended for readers to comment and shed light on specific manuscripts or topics of the day, or for editors to reply to questions raised by readers, particularly students. Contributions to Correspondence will be published at the discretion of the editors. I am excited about launching this section. Our community currently does not have a forum such as this, and I believe that there are some important issues that could benefit from an open exchange of ideas.

**How does the *PLoS Genetics* editorial process operate?** The *PLoS Genetics* editorial team is drawn from the community it serves. As a result, all review—whether by associate editors assigned to papers or the peer reviewers they invite—is conducted by experts in a broad range of disciplines. Reviewers are instructed to be thorough and decisions are reached after consultation between associate editors and myself.

The 30 or so members of the *PLoS Genetics* Editorial Board serve as our associate editors. They are a talented and dedicated group who handle each paper individually, but they are also encouraged to work together. As is appropriate for a journal that has the community in mind, associate editors may consult with one another at any point during the review process using the journal's Web-based review system. I have observed so far that these exchanges are learned and insightful, and ultimately lead (I hope) to the fairest possible decision about a manuscript that still keeps the ambitious goals of *PLoS Genetics* in mind.

I also invite “guest editors” to oversee the review process for particular manuscripts when the expert on our board is either unavailable or has a conflict of interest with a submission, or when a manuscript simply “falls between the cracks” of our expertise. The guest editor feature reflects, in a way, the fact that we are a community journal. Some of you will be tapped for this sooner or later—consider yourselves warned!

Although we aim for high-caliber peer reviews, we also strive to keep the turnaround time as short as possible. Our goal is to return a response to authors within 30 days of submission, and we're off to a decent start. In just six months of submissions, our average turnaround time from receipt to first decision was 35 days.

**How are things going so far?** The response to *PLoS Genetics* has been greater than we imagined—well over 100 research articles submitted, more than 3,000 sign-ups for electronic table of contents (eTOC) alerts, and a steady stream of presubmission inquiries. Are we receiving the breadth of manuscripts we hoped for? Absolutely. One look at our July issue table of contents will show you studies that represent a variety of organisms (yeast—two kinds—plants, nematodes, flies, mice, cats—big and little—and humans) and areas of research (genome annotation, genetics, epigenetics, microarrays, cancer, glaucoma, neurodegeneration, and evolution). The August issue will add prokaryotes, fish, and other new dimensions to the journal. Several studies consider multiple organisms, matching well our goals of connecting the greater genetics and genomics community.

**What is the vision for *PLoS Genetics?*** The editorial team has guided the journal from an idea to the launch issue you see here, but what we become is ultimately determined by the will and participation of the genetics and genomics community. I hope that you will embrace and, in one way or another, become a part of *PLoS Genetics*.  █

